# Effects of Yogic Interventions on Patients Diagnosed With Cardiac Diseases. A Systematic Review and Meta-Analysis

**DOI:** 10.3389/fcvm.2022.942740

**Published:** 2022-08-04

**Authors:** Sheetal Kalra, Mohammad Miraj, Puneeta Ajmera, Riyaz Ahamad Shaik, Mohamed K. Seyam, Ghada M. Shawky, Sharifa M. Alasiry, Elsayed H. Mohamed, Hatim M. Alasiri, Msaad Alzhrani, Ahmad Alanazi, Mazen Alqahtani, Abdul Raheem Shaikh, Mohammad Lafi Al-Otaibi, Shakir Saleem, Sajjan Pal, Vineet Jain, Fuzail Ahmad

**Affiliations:** ^1^School of Physiotherapy, Delhi Pharmaceutical Sciences and Research University, New Delhi, India; ^2^Department of Physiotherapy and Health Rehabilitation, College of Applied Medical Sciences, Majmaah University, Al Majmaah, Saudi Arabia; ^3^School of Allied Health Sciences, Delhi Pharmaceutical Sciences and Research University, New Delhi, India; ^4^Department of Community Medicine/Public Health, College of Medicine, Majmaah University, Al Majmaah, Saudi Arabia; ^5^Department of Nursing, College of Applied Medical Sciences, Majmaah University, Al Majmaah, Saudi Arabia; ^6^Department of Physical Therapy, Buraydah Private Colleges, Buraydah, Saudi Arabia; ^7^Department of Internal Medicine, Security Forces Hospital, Riyadh, Saudi Arabia; ^8^College of Applied Medical Sciences, Al Maarefa University, Riyadh, Saudi Arabia; ^9^Department of Orthopedic Surgery, College of Medicine, King Khalid University, Abha, Saudi Arabia; ^10^Department of Public Health, College of Health Science, Saudi Electronic University, Riyadh, Saudi Arabia; ^11^Faculty of Physiotherapy, SGT University, Gurugram, India; ^12^Mewat Engineering College, Nuh, India

**Keywords:** cardiac diseases, yoga, cardiovascular outcomes, psychosocial outcomes, review, meta-analysis

## Abstract

**Objective:**

Presently, evidence-based research studies on the efficacy of complimentary therapies like yoga for patients with different cardiac diseases are limited and conflicting. The objective of this study is to conduct a systematic review and meta-analysis of randomized controlled trials (RCTs) on yogic interventions compared with usual care or non-pharmacological treatment in patients diagnosed with cardiac diseases.

**Methods:**

We conducted an electronic search of literature published from 2006 to May 2021 through five databases. PRISMA statement was used to develop and report a systematic review and meta-analysis protocol. Sixteen RCTs were included in the systematic review and 11 RCTs were used for meta-analysis. Outcome measures were blood pressure, lipid profile, and psychosocial measures. The Cochrane collaboration tool was used to assess bias risk.

**Results:**

The results show that yogic interventions resulted in significant reduction in systolic (d = 046; 95% CI.08–0.84; I^2^ = 81.86%) and diastolic blood pressures (d = 0.56; 95% CI.13–0.99, I^2^ = 84.84%). A medium statistically significant increase in HDL (d =0.67; 95% CI 0 to 1.33; I^2^ 79.7%) and a low but significant effect on LDL (d = 0.23; 95% CI −0.08–0.54; I^2^ 32.61%), total cholesterol (d =0.28; 95% CI −0.14–0.7; I^2^ 63.72%), and triglycerides (d = 0.43; 95% CI −0.1–0.97; I^2^ 76.64%) were observed. Pooled effect sizes showed a medium to low statistically significant effect on psychosocial indicators *viz*., QoL, stress, anxiety, and depression.

**Conclusion:**

The meta-analysis found strong evidence of effectiveness of yogic interventions on lipid profile, blood pressure, and psychosocial outcomes in patients with diagnosed cardiac diseases.

## Introduction

Cardiac diseases constitute a global public health challenge and a substantial cause of morbidity and mortality (1). Around 17.8 million people died of cardiac diseases across the world in 2017 that corresponds to 35.6 million years lived with disability and 330 million years of life lost (2, 3). Economic loss resulting from debilitating and mortal outcomes of various cardiac diseases has resulted into billions of dollars spent for healthcare and reduced work productivity (4). Although the progress in medical treatment has resulted into a reduction in the rate of mortality resulting from different cardiac diseases, these are still the most important cause of death worldwide (5).

The United Nations in 2011 set out an aspiring plan to conclusively reduce the effect of non-communicable diseases. In addition to pharmacological treatment, investigations exploring the effects of complimentary therapies on overall management of cardiac diseases are getting equal importance. There is a growing body of evidence that supports that teams associated with rehabilitation of patients with cardiac diseases are exploring more and more non-traditional methods of interventions like mind-body interventions, dance and hydrotherapy, and music (6–8). Yoga can be defined as a holistic approach to mind body interaction and is an amalgamation of various physical postures, respiratory training, and meditation practices. The word “yoga” is derived from a Sanskrit word that is metaphorically described as the association of inner self with the universe with a main purpose to achieve consonance between the mind, body, and soul. Its eventual aim is to achieve salvation of the soul (9). Yoga also incorporates modifications in lifestyle habits such as diet control and abstention from smoking and alcohol (10). Various research studies in recent past have supported the fact that yogic interventions have numerous physical and psychological health benefits by downregulating the hypothalamus-pituitary-adrenal axis and the sympathetic component of the autonomic nervous system. Various reported benefits of yoga include reduction in blood pressure, enhanced dominance of the parasympathetic nervous system over the sympathetic nervous system, normalization of endocrinal function and gastrointestinal function, improved musculoskeletal fitness and posture, enhanced energy levels, normalization of body weight, better sleep and immunity, reduced pain, and better quality of life, whereas psychological benefits associated with yoga include enhanced mood, feeling of subjective well-being and self-acceptance, and reduced anxiety and depression (11, 12). The literature also reports improved biochemical profile in terms of antioxidant effects of yoga like reduced cholesterol, triglycerides, and glucose levels, increased lymphocyte count, and many more (13, 14).

## Rationale

The last few years have seen increased attention being paid to yogic interventions by the medical community for management of various medical conditions. This has happened because of increased acknowledgment and comprehension related to yoga. Effects of yogic intervention on modifying risk factors related to cardiac diseases and rehabilitation are continuously gaining significant importance (15). Moreover there is enough evidence to prove that yogic interventions are safe, effective, and can be used as compliment to pharmacological management and have also been found successful for treating various conditions such as sleep disorders, chronic pain syndrome, hypertension, post-menopausal syndrome, and diseases related to the cardiovascular system (5, 16). Evidence also supports the role of yoga in modifying various risk factors associated with cardiovascular diseases (CVDs) such as diabetes, obesity, psychological stress, and hypertension (17–21). The positive results underpin the inclusion of mind-body interventions in management of patients with cardiac diseases.

At present, evidence-based research studies on the efficacy of mind-body interventions like yoga therapy in patients with different cardiac diseases are limited and conflicting. Most of the research studies have focused on comparative studies on different types of training. Reviews on articles conducted previously have found yogic interventions feasible in patients with various diagnosed cardiovascular diseases. Previous reviews and meta-analysis have recommended a requirement of high-quality studies related to yogic interventions for patients with various cardiac diseases. This study extends a previously conducted study with focus on randomized controlled trials incorporating the impact of yoga interventions on anthropometric dimensions and different physiological parameters like mental health and cardiovascular variables, and psychosocial parameters like quality of life, stress, anxiety, and depression in patients diagnosed with cardiac diseases.

### Objective

The objective of this study is to conduct a systematic review and meta-analysis of the effects of yogic interventions on patients diagnosed with cardiac diseases focusing on physical and psychosocial outcomes.

## Methods

### Eligibility Criteria

Studies fulfilling the following eligibility criteria were included:

(a) Randomized control trial as the study design. (b) Full-text articles written in English, diagnosed cardiac condition, yogic intervention compared to or in addition to standard treatment (routine pharmacological treatment/cardiac rehabilitation/educational sessions/physiotherapy based lifestyle modifications) or no treatment at all in adult patients with cardiac diseases. (c) Full-text studies that examined physical outcomes including cardiovascular-related health parameters, anthropometric measurements, inflammatory markers, antioxidant status, and hemodynamic parameters, and psychosocial outcomes like anxiety, stress, and depression.

Studies that were excluded were those that included yoga as part of a mind-body intervention program, review articles, or meta-analysis, abstracts, opinion articles or letter to editors, and interventions other than randomized control trials.

**Figure 1:**
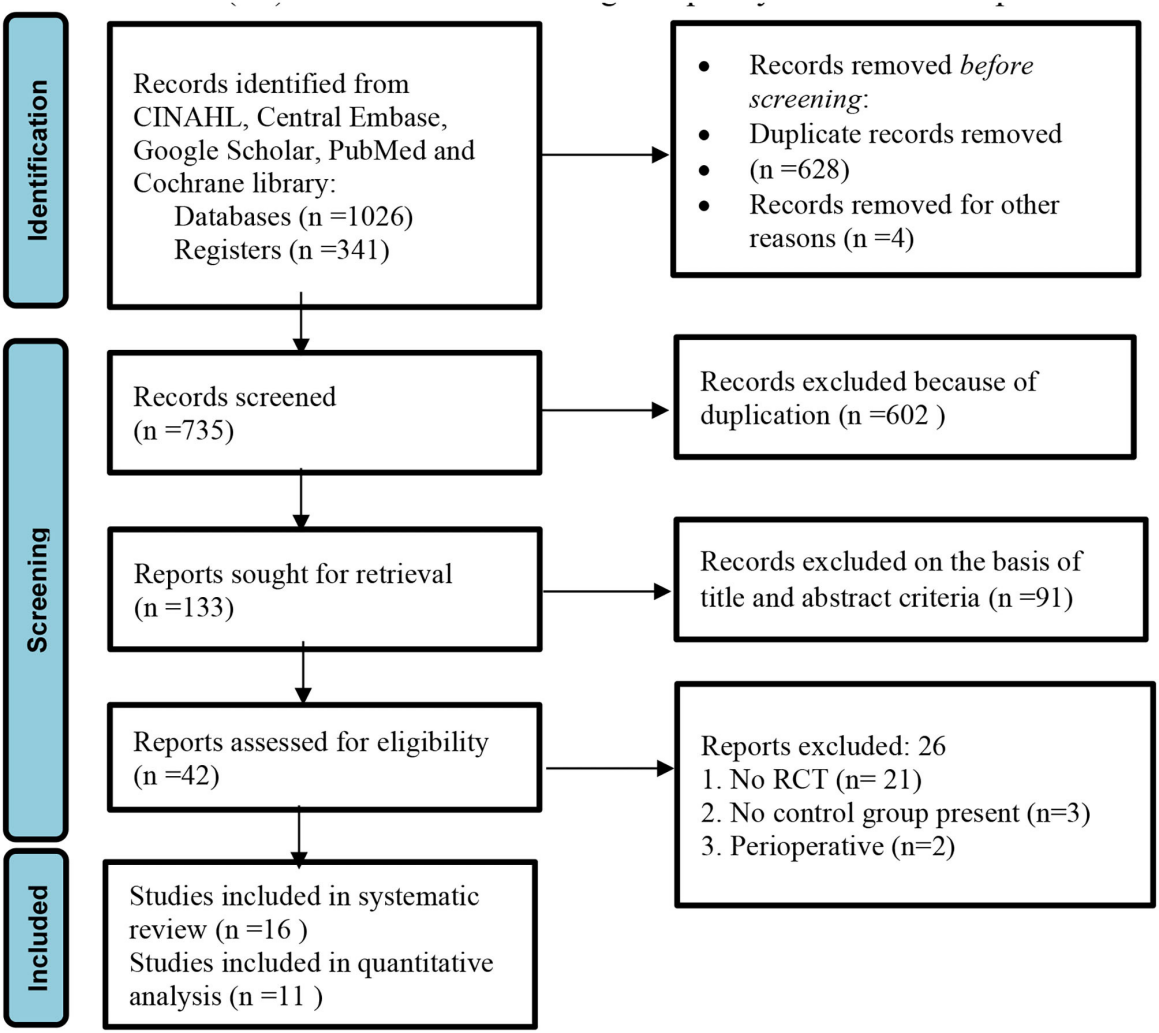
PRISMA flow diagram of the study (22).

The PRISMA flow diagram of study is presented in Figure 1.

### Information Sources

We conducted an electronic search of literature published in last the 15 years, i.e., from 2006 to May 2021 through five databases, *viz*., CINAHL, Central Embase, Google Scholar, PubMed, and Cochrane Library. Randomized controlled trials (RCTs) on yogic interventions compared with usual care or non-pharmacological treatment in patients diagnosed with cardiovascular diseases were included.

### Strategy for Literature Search

To optimize the search strategy and to make the search more precise, MeSH terms and Boolean operators were used in library databases. The search strategy used was: [“yoga OR yog OR yogic practices OR yogic interventions] AND [cardiac diseases OR cardiovascular diseases OR cardiac failure OR heart failure OR heart attack OR hypertensive heart disease OR atrial fibrillation OR ischemic heart disease OR valvular heart disease OR cardiomyopathy”]. Potential randomized controlled trials that met the inclusion criteria of the study were also screened by searching references of selected articles. The preferred reporting items for systematic review and meta-analysis protocols (PRISMA) statement was used to develop and report systematic a review and meta-analysis protocol (23).

### Data Extraction and Management

Data were independently extracted by two reviewers, SK and PA, on characteristics of study location, year of study, participants, study duration, sample size, male-female ratio, inclusion and exclusion criteria, details of intervention given to experimental groups and control groups, duration of study, outcome measures, and results of study. The data were rechecked by a third reviewer, SP, and all disagreements and discrepancies were resolved by consensus.

### Effect Measures

Outcome measures were blood pressure, lipid profile, and psychosocial measures. The Cochrane collaboration tool was used to assess bias risk. For analyzing maximum treatment effect, post-intervention mean scores and standard deviations related to pre-defined outcome measures were used in order to pool the data. In studies where different scales were used to measure the outcome, effect size (standardized mean difference, d) was calculated.

### Risk of Bias in Individual Studies

Risk of bias in individual studies and methodological quality assessment was performed by the 2 independent reviewers, SK and PA, with more than 15 years of experience in empirical research. The Cochrane collaboration tool was used to assess bias risk in randomized control trials in selected articles. The tool assesses bias risk on the basis of 7 domains. Judgment regarding bias was categorized into 3: (a) low risk, (b) high risk, and (c) unclear risk. PRISMA guidelines were used for reporting the results of systematic reviews and meta-analysis. Any disagreement between the 2 reviewers regarding appraisal recommendation was resolved by another reviewer (VJ). The results of methodological quality assessment are presented in **Table 2**.

### Data Synthesis and Meta-Analysis

Post intervention mean score and standard deviation of outcome measures were used for pooling the data. Effect size (standardized mean difference, d), whenever required, was calculated by subtracting the mean of post intervention score of the control groups from that of the yoga group. The result was divided by pooled standard deviations of both groups. An effect size of.2–0.5, 0.5–0.8 and more than 0.8 is considered small, moderate, and large, respectively (Cohen). A positive value of SMD indicates beneficial effects of yoga in comparison to control and vice versa. Also, in three studies, median and range were reported instead of post intervention mean score/standard deviation and mean differences. Hogg's formula was used in these cases to calculate post intervention mean scores and standard deviation (38, 39).

The Meta-Essentials software was used for summarization of data for all the outcome measures provided that at least two studies were available on a particular outcome. Random-effects models were used to calculate summary estimate with 95% confidence interval. A random-effects model is based on the assumption that selected studies were drawn from a population of studies that are systematically different from each other. Forest plots were constructed to graphically display the results. Zero specifies no heterogeneity, 25% is low, 50% is moderate, and 75% is high heterogeneity. A value of more than 0.05 in Q statistic indicated variance in studies and not between studies. An analysis was conducted on all the studies with exclusion of outliers.

## Results

### Study Selection Search Results

In the initial literature search, 1,367 titles emerged as relevant studies. After removal of duplicates and studies not the fulfilling eligibility criteria, a total of 42 unique full-text records were identified. A total of 15 RCTs were included in the qualitative analysis. Four RCTs reported different outcome measures and were excluded from the quantitative analysis (meta-analysis). Eleven RCTs with sufficient data were included for the meta-analysis (Figure 1).

### Study Characteristics

Out of the total 15 randomized controlled trials included in this review, six were conducted in India, places of 16 RCT are mentioned, two in Atlanta, two in Sweden, one each in Brazil, the United Kingdom, Tehran, and Poland; one was a collaboration between India and the United Kingdom. Five RCTs included patients with heart failure, four with coronary artery disease, three with acute myocardial infarction, and one with atrial fibrillation. Two RCTs were performed on post CABG (coronary artery bypass graft) patients. The study by Fathollahi, Raghuram, and Grabara had male participants only (24, 26, 33), while the study by Pal (34) did not mention about gender. Tillin et al. did not mention about the exclusion criteria of the study (27). Most of the studies mentioned intervention provided to a yoga group by a certified yoga therapist/experienced yoga instructor. The study by Christa et al. and Fathollahi et al. did not mention about the instructor (26, 28). All the trials were conducted either in the hospital (yoga clinic, physiotherapy department) or in a medical institute/university. Details of study population are presented in Table 1. All yoga trials were composed of yogic postures and breathing, training, and relaxation exercises. Duration, frequency of yoga sessions, and length of entire study varied among the trials. Yoga session duration ranged from 30 to75 min, with 16 as maximum total sessions. Yoga length, frequency, and duration varied between the trials. Treatments extended to control groups included standard care in the form of cardiac rehabilitation, pharmacotherapy, educational advice, or lifestyle modification. Baseline measurements were provided in all the RCTs. The time of assessment of outcome measures varied from 24 days, 12 weeks, 13 weeks, 24 weeks, 1 year, to 5years. Studies by Pal et al. did not describe about home sessions to the patients (34, 35).

**Table 1:** Description of study populations.

**References**	**Study** **Location**	**Diagnosis**	**Sample size** **(baseline and follow** **up)**	**Age range/mean** **age/Gender** **distribution**	**Eligibility criteria**	**Setting**
Prabhakaran et al. (10)	India, UK, Sponsored by ICMR, Medical Research Council UK	Acute MI,	Yoga care group *n* = 1,970 Enhanced standard caregroup *n* = 1,989 **Follow up** Yoga group *n* = 1,953 Control group *n* = 1,968	Age range−18–80 years **Mean age–** Yoga group- 53.4 ± 11.0 Control group– 53.4 ± 10.8 **Gender** Yoga group Females ± 13.8% Standard care group ± 14.1%	**Inclusion criteria**–Patients with acute MI in past 14 days, age 18–80 years, willing and able to complete. **Exclusion criteria–** Patients who practiced yoga regularly>3 h per week, participating in other clinical trials, had diseases that limited their life span to <1 year, considered unlikely to complete study.	Trials performed at CDC, Hospital based
Grabara et al. (24)	Poland	ST elevation, Myocardial infarction	Total−70 male patients	Age range−45–65 years **Mean age-** Yogagroup- 57.1 ± 5.3 Control group- 49.6 ± 5.03 **Gender**-Males only	**Inclusion criteria-** Patients with uncomplicated STEMI enrolled between first and second months after STEMI. Patients ≤ 70 years, stable with respect to symptoms and medication. **Exclusion criteria-** unregulated hypertension, unstable angina, arrhythmias, conduction disorders varicose veins, unhealed injuries of lower limbs, advanced peripheral atherosclerosis, diagnosed cancer diseases of central and peripheral nervous system, LVEF <35%, stress test results obtained before CR programme, ≤ 7 METS, post-operative complications that limited improvements incomplete medical documentation.	
Sharma et al. (25)	India	Coronary artery disease	Yoga group *n =* 33 Control group *n =* 33 **Follow up** Yoga group *n =* 32 Control group=32	Age range-30–65 years **Mean age-** Control group−51.51 ± 81.5 Yoga group−53.15 ± 11.59 **Gender** Yoga group– Men −26, Women-7 Control gp-Men−31, Women−2	**Inclusion criteria–** Recent MI on conservative medical treatment without involving revascularization procedures from 10 days−2 months post MI, Left Ventricular dysfunction NYHA, Class I and II LVEF between 30 and 50%. **Exclusion Criteria–** LVEF <30%,class III and IV heart failure, unstable cardiac symptoms like angina recurrent ischemia, COPD, Uncontrolled arrhythmia, severe musculoskeletal problems that limited participation in yoga, hypertension SBP>160 mm of Hg, DBP>100 mm of Hg, valvular heart disease patients with hematological, renal or hepatic dysfunction.	Hospital based yoga Center
Fathollahi et al. (26)	Tehran	Post CABG	Yoga group *n =* 10 Control group *n =* 10 **Follow up** YCRT *n =* 7 CRT *n =* 7	Age range 40–75 years **Mean age** Yoga group– 61.10 ± 10.66 Control group– 64.3 ± 10.57 **Gender** Males only	**Inclusion criteria–** EF>35%,no experience for emergency heart surgery, lack of motor disorder, yoga training experience, associated illness. **Exclusion criteria–** lack of follow up and regular attendance at training sessions, changes in patients echo and increasing of chest pain.	Medical&Research Center
Tillin et al. (27)	UK	Acute coronary Event	Yoga+Usual care group *n =* 40 Usual caregroup *n =* 40 **Follow up** Yoga gp−25 Control gp−35	Age range- 35–80 years **Mean age** Yoga group−57.4 ±3.3 Control group– 56.9 ± 3.1 **Gender** Yoga+Usual care group Men−70% Women−30% Usual care group Men−67% Women 23%	**Inclusion criteria–** referral to cardiac rehab program following acute coronary syndrome, age 35–80 years, male–female without comorbid conditions or mobility limitations, able to understand English or Punjabi, patients who have undergone coronary artery bypass grafting or who had received. **Exclusion criteria-** not mentioned.	Hospital and primary care centers
Christa et al. (28)	India	Acute MI	Yoga group *n =* 40 Standard care *n =* 40 **Follow up** -100%	Age range-18–65 years **Mean age** Yoga group- 49.88 ± 9.36, Control group- 50.00 ± 9.22 **Gender** Yoga group Men- 94.87% Standard care group Women- 95%	**Inclusion criteria-** both men and women I the age group of 18-65 years with acute MI within the previous month willing to participate and attend complete hospital based cardiac rehabilitation programme, patients who had undergone revascularization surgery. **Exclusion criteria-** presence of any disease that limited life expectancy less than 1 year, already participating in yoga programme> 3 hrs./week Patients participating in any other clinical trials.	Hospital based
Prasad et al. (29)	Brazil	Heart failure	Yoga group *n =* 100 Control group *n =* 100 **Follow up** -100%	Age range 45–75 years **Mean age**-60 ± 11.53 years Yoga group 59.83 ± 11.41 Control group 60 ± 11.53 **Gender** Women-68, Men-32 in both groups	**Inclusion criteria-** more than 18 years of age, patients with MI. **Exclusion criteria-** hemodynamically unstable patients, advanced heart failure, pregnancy, post-partum <3 months, practiced yoga>1 time per month in last 6 months, known arrhythmias, on cardiac pacemaker, autonomic neuropathy musculoskeletal problems that limited participation.	National Heart Institute
Hagglund et al. (30)	Sweden	Heart failure	Hydrotherapy *n =* 20 Yoga *n =* 20 **Follow up** Hydrotherapy *n =* 12 Yoga *n =* 18	Age range- 18–80 years **Mean age** Yoga group- 64.1 ± 9.4 Hydrotherapy group- 65.7 ± 8.5 **Gender** Hydrotherapy group Women-9 Yoga group Women-5	**Inclusion criteria-** age range 18-80 years, Heart failure of ischemia or non-ischemia etiology diagnosed to European society of cardiology guidelines,NYHA class I-III **Exclusion criteria-** Patients with dementia, with life expectancy less than 6 months, scheduled for heart transplantation, unable to understand verbal. Instructions, patients with incontinence, addicted to alcohol or drugs, had wounds or allergic to chlorine.	Hospital based
Wahlstrom et al. (31)	Sweden	Atrial fibrillation	Yoga group *n =* 40 Control group *n =* 40 **Follow up** Yoga group *n =* 33 Control group *n =* 36	Age range-Not mentioned Yoga group- 64 ± 7 Control group- 63 ± 8 **Gender** Yoga group Women-17 Control group Women-26	**Inclusion Criteria-** Diagnosed Paroxysmal Atrial fibrillation necessitating pharmacological management on medical treatment for at least 3 months. **Exclusion criteria-** Difficulty in understanding Swedish language, patients with multiple concurrent medical conditions, cognitive dysfunction.	Hospital based (Physiotherapy clinic)
Krishna et al. (32)	India	Heart failure	Yoga group *n =* 65 Control group *n =* 65 Yoga group Control group-33.33% **Follow up** Yoga group *n =* 44 Control group *n =* 48	Age range-Not mentioned Yoga group- 49.34 ± 5.70 Control group- 50.14 ± 4.54 Follow up-100% **Gender** Yoga group Men-32, Women 12 Control group Men-32, Women-16	**Inclusion criteria-** Patients who had systolic or diastolic dysfunction, ejection fraction of <30% who satisfied New York Heart Association Class I–II, able to walk with no assistance, on stable medical therapy. **Exclusion criteria-** Patients with COPD, unable to attend Yoga classes, orthopedic impediments to Yoga hospitalized in a last 3 months, suffered from MI or recurrent angina in last 3 months.	Medical Education & Research Institute
Raghuram et al. (33)	India	Post CABG	Yoga group *n =* 129 Control group-121 **Follow up** Yoga group *n =* 89 Control group *n =* 76	Age range-35–65 years Yoga group- 53.34 ± 6.42 Control group- 52.6 ± 6.85 Gender-males only	**Inclusion criteria-** Those with established triple or double vessel disease, males between 35–65 years, those residing within 200 kms of hospital. **Exclusion criteria**- Emergency CABG, CABG with valvular surgeries, acute or chronic renal failure, with or without dialysis, physical disabilities that would prevent yoga practice, neuropsychiatric illness, patients already exposed to Yoga, LVEF <30%.	Hospital based
Pal et al. (34)	India	CAD	Yoga group-129 Control gp-129 **Follow up** Yoga group-105 Control group-103	Age range 35–82 years Yogagroup- 59.1 ± 9.9 Control group- 56.4 ± 10.9 Gender-Not mentioned	**Inclusion criteria-** Patients with proven CAD, willing to participate. **Exclusion criteria-** Patients with other comorbid conditions, patients with known complications of CAD, those on pacemakers and had undergone bypass surgery.	Medical University
Pal et al. (35)	India	CAD	Yoga group *n =* 85 Control group *n =* 85 **Follow up** Yoga group *n =* 80 Control group *n =* 74	Age range-40–75 years Yogagroup– 58.9 ± 9.4 Control group– 58.6 ± 10.5 **Gender** Yoga group Men−72, Women−13 Control group Men−72, Women−13	**Inclusion criteria-** Patients with clinical history of angina, ECG changes of ischemic heart disease treadmill positivity, history of MI and <70% narrowing of more than 1 or major coronary artery age 40-75 years. **Exclusion criteria-** patients with co-morbid conditions like diabetes mellitus, malignant hypertension, COPD, asthma, diseases of nervous system, endocrinal disorders, congenital heart disease, patients with known complications of CAD, AV block, on pace maker undergone bypass surgery.	Cardiology department, Medical Institute
Pullen et al. (36)	Atlanta	Heart failure	Yoga *n =* 21 Control *n =* 19 **Follow up** Yoga *n =* 18 Control *n =* 16	Age range 31–76 Yoga group– 55.8 ± 7.6 Control group– 52.5 ± 12.7 **Gender** Yoga group Men– 10, Women−11 Control group Men−13, Women−6	**Inclusion criteria–** Patients with systolic heart failure EF <45%or diastolic heart failure EF>45% of ischemic or non-ischemic etiology New York Heart Association (NYHA), Class 1–111 were able to walk without assistance, on stable medical therapy for at least 3 months before enrolment. **Exclusion Criteria–** Unable to walk without assistance, had life expectancy <6 months, were pregnant or breast feeding, unable to attend yoga classes twice a week addicted to alcohol or drugs.	Hospital based
Pullen et al. (37)	Atlanta Georgia	Heart failure	Medical treatment +Yoga (*n =* 9) Medical treatment (*n =* 10) **Follow up** −100%	Age range 31–76 Yoga group−52.1 ± 3.3 Control group−50.5 ± 12.8 **Gender** Yoga group Men−2, Women−7 Control group Men−7, Women−3	**Inclusion criteria–** Systolic Heart failure with LVEF <50% within 6 months before enrolment, New York Heart Association, class 1–111 were able to walk without assistance, on stable medical therapy for at least 3 months before enrolment. **Exclusion Criteria-** Unable to walk without assistance, had life expectancy <6 months, were pregnant or breast feeding, unable to attend yoga classes twice a week addicted to alcohol or drugs.	Hospital based

*MI, myocardial infarction; CDC, Center for Diseases Control; MET, metabolic equivalent STEMI, ST-elevation myocardial infarction; LVEF, left ventricular ejection fraction; CABG, coronary artery bypass graft; SBP, systolic blood pressure; DBP, diastolic blood pressure; NYHA, New York Health Association; CAD, coronary artery disease; ECG, electrocardiogram, COPD, chronic obstructive pulmonary disease*.

Table 2 presents a description of interventions provided to both groups, time of evaluation, and results obtained.

**Table 2:** Description of yoga program and attendance to classes.

**References**	**Yoga program (Y); Duration** **and frequency (D); Home practice (H) vs.** **comparison (C)**	**Instructor**	**Control group**	**Evaluation and result**
Prabhakaran et al. (10)	**Yoga**- First 2 individual sessions rest group sessions at hospital which included Pranayama breathing exercises, meditation and relaxation practice in supine and seated position followed by discussion on lifestyle modification. **Duration**- 75 min per session for 12 weeks **Home session**-After 2 weeks of monitored session patients practiced same procedure for 3 days at their homes **Total sessions** -13	Trained yoga teacher	enhanced standard care group received 3 sessions of educational advice with leaflet	**Evaluation-**baseline and 12th week **Result**-Yoga improved self- rated health and return to pre infarct activities but did not show any statistically significant improvement in major adverse cardiac event
Grabara et al. (24)	**Yoga**-Hathayoga consisted of Yogasanas, breathing exercises and pranayama **Duration**- 24 days of training per patient, total duration of study- 3 months, 60 min per session **Home sessions**- yoga practiced for 10 weeks	Yoga instructor	Standard Cardiac rehabilitation	**Evaluation-**baseline and 24th day **Result**-Addition of Hathayoga to Cardiac Rehabilitation resulted in improved cardiac hemodynamic parameters of patients with cardiac diseases compared to standard cardiac rehabilitation
Sharma et al. (25)	**Yoga**-supervised yoga module comprising of yoga asana, pranayama, breathing training, relaxation **Duration**-1 h thrice a week for 12 weeks **Home session**-Not mentioned	Certified yoga therapist with minimum qualification MSc yoga with minimum experience of 3 years in yoga	Standard pharmacological treatment and instructions from cardiologist	**Evaluation-**baseline and 12 week **Result**-Addition of yoga did not bring any significant improvement in cardiac function
Fathollahi et al. (26)	**Yoga**-Yogaasanas and pranayama **Duration**- 1 hthrice a week for 8 weeks **Home session**-Not mentioned	Not mentioned	Cardiac Rehabilitation	**Evaluation**-Baseline and 8th week **Result**-Yoga training may be added into conventional cardiac rehabilitation of patients with Coronary Artery Bypass grapy
Tillin et al. (27)	**Yoga**-breathing exercises, yoga postures and meditation and education and discussion **Duration** – 75 min twice weekly for 12 weeks **Home sessions**-prescription of exercises along with DVD provided to practice at home	Teacher certified in yoga and cardiac rehabilitation	lifestyle modifications which included Physical activity, diet, weight management, smoking cessation. Education regarding risk factormanagement	**Evaluation**-baseline and 3rd month **Result**-No discernible improvement seen with addition of yoga to standard cardiac rehabilitation programme
Christa et al. (28)	**Yoga**-There were 4 phases of yoga intervention. Educational advice was given during week 1 (Session 1), individual face to face yoga session was given in week 3(Session 2), Face to face group sessions of yoga and education was given in weeks 5–7(sessions 3–8 were given twice weekly between weeks 5&7 post Myocardial Infarction), similar session was given in week 8–13 once weekly (sessions 9–13). Yoga interventions included health rejuvenating exercises for 10 mins, yoga asanas for 25 mins in standing, sitting and lying poses, pranayama for 15 min, meditation and relaxation techniques for 15 min and discussion for 10 min **Duration**- 75 min for 12 weeks **Home sessions**-Not mentioned **Total sessions**-13	Not mentioned	Enhanced standard care received educational advice with leaflet before discharge(session1) followed by 2 similar educational sessional at week 5 and 12(session 2&3)in addition routine pharm treatment and surgical intervention	**Evaluation**-baseline and 13th week **Result-**Addition of yoga to cardiac rehabilitation programme improved cardiac autonomic functions with parasympathetic functions
Prasad et al. (29)	**Yoga**-10 mins of pranayama, 15 min of yoga asanas, 15 min of transcendental meditation **Duration**-40 min twice daily for 24 weeks **Home sessions**-not mentioned	Under supervision of clinician/resident/trained yoga expert	Conventional treatment and lifestyle counseling	**Evaluation**- baseline and 24th week **Result**–Addition of yoga with standard treatment and lifestyle modification resulted in significant improvements in primary and secondary outcomes.
Hagglund et al. (30)	**Yoga**–Breathing exercises, yoga posture, relaxation and meditation **Duration**−45–60 mins twice a week for 12 weeks **Home session**– once a week at home also. Mediyoga CD's and hand–outs of yoga postures given to patients	Certified yoga instructor Certified physical therapist	45 min hydrotherapy session in heated therapy pool twice a week for 12 weeks	**Evaluation**–Baseline and 12^th^ week **Result**–Yoga may be considered as complementary to standard hydrotherapy treatment in improving quality of life and reducing depression in patients with heart failure
Wahlstrum et al. (31)	**Yoga**–Kundalini yoga which consisted of deep breathing exercises for 5–10 min followed by 2 breathing techniques, meditation for 10 min and relaxation 10 min. **Duration**– 1 hour once a week for 12 weeks **Home sessions**–Participants received written programme that included movement descriptions ad a CD. yoga was conducted at median of 2(range 1–4) sessions per week **Total sessions**−10, average (8–12)	Experienced yoga instructor	Standard medication cardio version and cardio ablation was also given	**Evaluation– baseline and 12th week** **Result**–Addition of yoga to standard treatment led to improvement in quality of life, lower Blood Pressure,Heart rate
Krishna et al. (32)	**Yoga**–Pranayama breathing exercises, meditation and relaxation practice in supine and seated position **Duration–**60 mins 6 days a week for 12 weeks **Home sessions**–After 2 weeks of monitored session patients practiced same procedure for 3 days under direct supervision and 3 days at their homes	Yoga therapist with expertise in cardiac rehabilitation	standard medical therapy	**Evaluation**–baseline and 12th week **Result**–addition of yoga to standard medical therapy resulted into decrease in oxidative stress and inflammatory markers
Raghuram et al. (33)	**Yoga**– 1^st^ module (preop– 6 weeks) included deep relaxation technique, mind sound resonance technique, naddishudha pranayama Physical postures and pranayama added in 2^nd^(6 weeks−6 months) and 3^rd^ yoga module (6 months−12 months).Also included counseling on yogic lifestyle modifications **Duration**– 30 min session twice a day for 12 months **Home sessions**– 1 h practice per day monitored through lifestyle diary and weekly phone call	Yoga therapist	Physiotherapy based lifestyle modification programme pre op−6 weeks–breathing exercises, physiotherapy exercises for hand, elbow neck, back, legs 6 weeks−6 months–breathing exercises, additional exercises for above joints 6 months−12 months–Additional physiotherapy exercises	**Evaluation**–baseline, 6^th^ week, 6th month, 1 year **Result**–Addition of yoga to conventional cardiac rehabilitation resulted into better management of risk factors
Pal et al. (34)	**Yoga–**Yogic practices, **Duration**– 35–40 min a day, 5 days a week for 18 months **Home sessions**–Not mentioned	Yoga instructor(post graduate)	Medications	**Evaluation– baseline and 18th month** **Result–**A significant improvement was seen in patients that practiced yoga as adjunct in patients with CAD
Pal et al. (35)	**Yoga**–Jal neti, Asanas,Pranayama, Om chanting **Duration**−35–40 min per week, 5 days in a week for 6 months **Home sessions**–Not mentioned	Yoga expert faculty	Medications	**Evaluation–baseline and 6th month** **Result**–Yogic practices resulted in improved health of patients with cardiac diseases
Pullen et al. (36)	**Yoga**–Breathing exercises, hathayoga, meditation and relaxation **Duration**−1 h twice a week for 8–10 weeks Home sessions–handouts of 18 yoga postures given which they practiced with 3 sessions at home **Total sessions**−16 supervised sessions	Registered Yoga teacher	Standard medical therapy	**Evaluation–baseline and 8th week** **Result**–Yoga therapy along with standard care offered additional benefits in patients with heart failure
Pullen et al. (37)	**Yoga**–asanas, relaxation and breathing techniques, pranayama, meditation **Duration**−70 min session twice a week for 8 weeks **Home sessions**– hand–outs and videos given of 18 yoga postures which they practiced at–least 1 session at home **Total sessions**−16	Registered Yoga teacher	Standard medical therapy	**Evaluation–Baseline and 8th week** **Result–**Yoga improved exercise tolerance, positively affected inflammatory markers and improved quality of life in patients with heart failure

The number of dropouts varied in all the studies. The highest number of dropouts was reported in the study of Raghuram (40 from the intervention group and 45 from the control group) followed by the study of Prabhakaran (29 from the intervention group and 19 from the control group), Pal (34) (24 from the intervention group and 26 from the control group), Krishna (21 from the intervention group and 17 from the control group), Tillin (11 from the intervention group and five from the control group), Wahlstrum (seven from the intervention group and four from the control group), Hagglund (two from the intervention group and 8 from the control group), Prasad (two from the intervention group and six from the control group) Fathollahi (two from the intervention group and three from the control group), Pullen (three from the intervention group and 8 from the control group), and Sharma (one from the intervention group and 1 from the control group).

The main barriers for not completing the study include unwillingness to continue with yoga classes, participants frequently citing ill health as a reason, return to work, family issues, long travel distance to the hospital, muscular skeletal symptoms, cognitive decline, and constraints in time to come for follow-up.

### Risk of Bias Assessment

Random sequence generation was conducted on all the 15 trials. Only four trials reported allocation concealment, while it was unclear in five trials reporting unclear risk of selection bias. Eleven trials did not report on blinding of patient and personnel and were therefore rated as high risk of performance bias. Seven trials did not report on blinding of outcome assessors, and four trials reported unclear risk of detection bias. Seven trials reported loss to follow-up, and four trials that were unclear about loss to follow-up were rated as unclear risk of attrition bias. The 15 trials reported all the outcomes and were assessed as low risk of selective reporting bias. A detailed description of the risk of bias assessment is presented in Table 3.

**Table 3:** Risk of bias assessment using the cochrane risk of bias tool.

**S.No**	**Study**	**Random** **sequence** **generation**	**Allocation** **concealment**	**Blinding of** **patient and** **personnel**	**Blinding of** **outcome** **assessment**	**Incomplete** **outcome data** **addressed**	**Selective** **reporting**	**Other Bias**	**Score**
	**Selection bias**		**Performance** **Bias**	**Detection** **Bias**	**Attrition** **bias**	**Reporting** **bias**			
S1	Prabhakaran et al. (10)	Yes	Yes	No	Yes	Yes	Yes	Yes	6
S2	Grabara et al. (24)	Yes	Unclear	No	No	No	Yes	Yes	3
S3	Sharma et al. (25)	Yes	Yes	Yes	Yes	Yes	No	Unclear	5
S4	Krishna et al. (32)	Yes	No	No	No	Unclear	Yes	Unclear	2
S6	Fathollahi et al. (26)	Yes	No	No	Unclear	Yes	Yes	No	3
S7	Christa et al. (28)	Yes	Unclear	No	No	Unclear	Yes	Unclear	2
S8	Tillin et al. (27)	Yes	Yes	No	Unclear	No	Yes	Unclear	3
S9	Prasad et al. (29)	Yes	NO	No	No	Unclear	Yes	No	2
S10	Haglund et al. (30)	Yes	No	No	No	Yes	Yes	No	3
S11	Wahlstrum et al. (31)	Yes	Yes	Yes	Unclear	Yes	Yes	Unclear	5
S12	Raghuram (33)	Yes	Unclear	No	No	Yes	Yes	Unclear	3
S13	Pal (34)	Yes	No	No	No	Yes	Yes	Unclear	3
S14	Pal (35)	Yes	Unclear	No	Unclear	Yes	Yes	Yes	4
S15	Pullen et al. (36)	Yes	No	Unclear	Yes	Unclear	Yes	Unclear	3
S16	Pullen et al. (37)	Yes	Unclear	Unclear	Yes	Unclear	Yes	Unclear	3

### Results of Syntheses and Intervention Outcomes

#### Physiological Outcomes

##### Resting Blood Pressure

**Figure 2:**
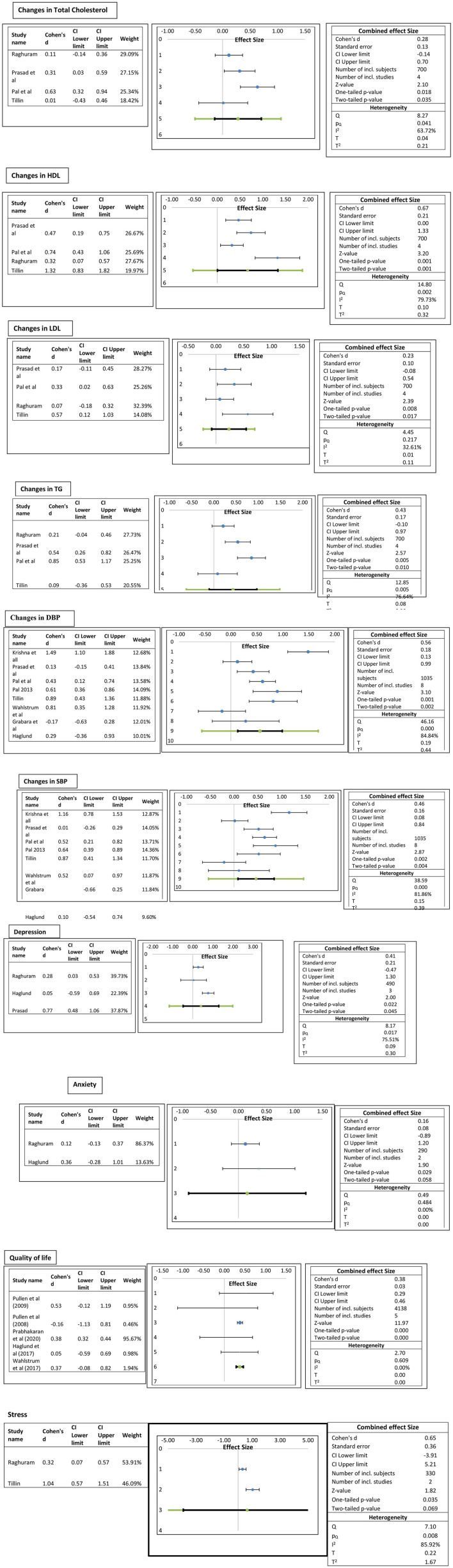
Forest plots of the effect of yogic interventions on physiological and psychosocial parameters.

Eight trials comprising 1,035 participants reported resting blood pressure. The results show that yogic interventions resulted in significant reduction in systolic blood pressure and diastolic blood pressure. Effect sizes for SBP and DBP depicted a similar heterogeneity pattern. The pooled results depict a low but statistically significant effect on SBP (Cohen d = 0.46; 95% CI.08–0.84; I^2^ = 81.86%) and a medium effect on DBP (Cohen d = 0.56; 95% CI.13–0.99; I^2^ = 84.84%) (Figure 2).

#### Lipid Profile

Four trials comprising 700 participants reported lipid profiles. The pooled results depict a medium statistically significant increase in HDL in the yoga group compared to the control group (Cohen d = 0.67; 95% CI to 1.33; I^2^ 79.7%) and a low effect on LDL (Cohen d = 0.23; 95% CI −0.08–0.54; I^2^32.61%). Similarly, a low effect on total cholesterol (Cohen d = 0.28; 95% CI.14–0.7; I^2^ 63.72%) and triglycerides (Cohen d = 0.43; 95% CI −0.10 to.97; I^2^ 76.64%) was observed (Figure 2).

#### Psychosocial Outcomes

It was observed that overall yoga had statistically significant promising results in psychosocial outcomes. Quality of life was assessed among 4,138 participants in five trials with three different questionnaires [Minnesota Living with Heart Failure Questionnaire, MLWHFQ], The Kansas City Cardiomyopathy Questionnaire (KCCQ), and EuroQol-5 Dimension (EQ-5D)]. The pooled effect sizes show medium statistically significant (Cohen d = 0.38; 95% CI 0.29 −0.46 to 0; I^2^ 0%) QoL score. It was observed that the results of the study by Prabhakaran et al. (10) were influenced by large sample size. Stress was measured in two studies with the same instrument, Perceived Stress Scale. The pooled results depict a medium statistically significant effect on stress scores (Cohen d = 0.65; 95% CI −3.91 to 5.21; I^2^ 85.92%). Anxiety was assessed with “The Hospital Anxiety and Depression Scale (HADS)” in two studies and depression in three studies with two scales, HADS and “Beck's Depression Inventory.” The pooled results showed a low statistically significant effect size of d = 0.16 (95% CI −0.89 to 1.2, I^2^ 0%) on stress and a medium statistically significant effect size of d =0.41 (95% CI −0.47–1.3, I^2^ 75.5%) (Figure 2). Description of physical and psychological outcomes of selected studies are shown in Table 4.

**Table 4:** Description of physical and psychosocial outcomes and between group differences (yoga vs. control).

**References**	**Setting**	**Between group** **differences**				**Psychosocial** **outcomes**			
Prabhakaran et al. (10)	Trials performed at Center of chronic disease control; hospital based		**Yoga care**	**Control group**	***p*** **value**				
		**Primary outcomes**							
		Major Adverse Cardiac Events	131 ± 6.7	146 ± 7.4	0.41				
		Self–related health	77.0 ± 16.8	75.7 ± 17.8	0.002				
		**Secondary outcomes**	**Yoga care**	**Control group**	***p*** **value**				
		Return to pre infarct activities	88.3 ± 18.9	87.0 ± 20.1	0.039				
		High medication adherence	1,199 ± 64.6	1,210 ± 64.3	0.88				
									
		Tobacco cessation	449 ± 76.2	445 ± 77.5	0.6				
									
		Other outcomes	77	77	0.95				
		Death from any cause	13	15	0.73				
		Non–fatal stroke	4	3	0.7				
		Death from any cause	13	15					
Grabara et al. (24)		**Cardiac hemodynamic parameters**	**Experimental group**	**Control group**	***p*** **value**				
		• LVEF%	48.8 ± 7.2	51.9 ± 4.7	0.0081				
		• LVEDD (mm)	50.6 ± 5.0	50.7 ± 1.8	NΣ				
		• LVESD (ms)	34.7 ± 2.7	35.1 ± 2.8	NΣ				
		**Spiroergometric stress test parameters**	**Experimental group**	**Control group**	***p*** **value**				
		• HR Rest bpm	61.7 ± 8.6	70.5 ± 7.2	NΣ				
		• HR Peak bpm	113.4 ± 11.2	131.6 ± 11.2	NΣ				
		• SBP rest, mm Hg	130.3 ± 14.8	127.7 ± 9.5	NΣ				
		• DBP rest,mm Hg	81.7 ± 8.0	80.6 ± 4.4	NΣ				
		• SBP,Peak,mm Hg	144.9 ± 21.5	154.6 ± 12.9	NΣ				
		• DBP,peak, mmHg	85.2 ± 6.2	84.6 ± 4.1	NΣ				
		• Test Duration(min)	7.7 ± 1.1	7.3 ± 1.0	0.00044				
		• MET,mlO2/kg/min	8.8 ± 0.7	7.9 ± 1.2	0.00048				
		• VO2 peak,ml O2/kg/min	37.4 ± 2.7	39.3 ± 3.8	3.0000				
Sharma et al. (25)	Hospital based yoga center	**Cardiac function**	**Yoga group**	**Control group**	***p*** **value**	**Improvement seen in**	**Yoga group**	***p*** **value**	
		LVEF	52.53 ± 0.832	50.9 ± 0.859	0.218	CDS	U= 71	0.00	
		Metabolic equivalent U= 136				HAM–S	U=128	0.00	
						QOL (DASI)	U=146	0.00	
Fathollahi et al. (26)	Medical &Research center	**Inflammatory biomarkers**	**Yoga group**	**Control group**	***p*** **value**				
		hsCRP(mg/L)	3.13 ± 2.03	2.00 ± 1.26	0.16				
		IL−6(pg/ml)	9.40 ± 2.87	8.77 ± 2.53	0.234				
Tillin et al. (27)	General hospital and primary care CR centers	**Primary outcomes**	**Yoga** **+usual care**	**Usual care**	***P*** **-value**	**Secondary outcomes**	**Yoga** **+usual care**	**Usual care**	**P value**
		LV diastolic function	8.81 (8.33 to 9.29)	8.26 (7.79 to 8.74)	0.4	**Resting blood pressure and heart rate**			
		6 Min walk test	488 (463 to 513)	491(471 to 512)	0.7	Brachial DBP, mm Hg	5 (73 to 76)	73 (71 to 74)	0.6
		3 Min step–test	82 (79 to 84)	79 (77 to 82)	0.4	Heart rate, bpm	59 (57 to 61)	61 (60 to 63)	0.8
						HPA axis, Walking	11.2 (8.7 to 13.7)	12.5 (10.4 to 14.7)	0.5
						Autonomic function, HRV	29 (6 to 53)	26 (2 to 49)	1
						Metabolic measures, LDL cholesterol, mmol/L	1.76 (1.66 to 1.87)	1.81 (1.71 to 1.92)	0.9
		**Time domain indices of HR variability**	**Yoga Group**	**Standard Care Group**	***p*** **-value**				
		Variance	320.13 (−3,315.16–10,435.50)	338.89 (−6,920.76–2,909.33)	0.883				
		SDNN (ms)	4.59 (−27.78–70.93)	5.98 (−51.38–24.23)	0.875				
		SDSD (ms)	1.74 (−80.38–96.76)	4.53 (−127.52–44.37)	0.899				
		RMSSD (ms)	1.73 (−80.22–96.44)	4.52 (−127.31–44.27)	0.891				
		NN50	3.00 (−21.00–203.89)	1.23 (−66.00–45.00)	0.307				
		pNN50 (%)	0.60 (−8.42–90.39)	0.41 (−23.59–19.53)	0.468				
		VLF (ms2)	107.37 (−2,950.94–10,662.06)	75.91 (−5,389.33–1,184.55)	0.59				
		LF (ms2)	51.26 (−1,305.10–3,542.77)	60.90 (−832.55–1,703.70)	0.829				
		HF (ms2)	114.42 (−794.80–7,993.78)	−38.14 (−4,843.50–1,617.87)	0.005				
		Total power (ms2)	261.85 (−3,898.18–13,496.96)	128.43 (−5,767.94–5,218.50)	0.984				
		**Frequency domain indices**							
		VLF (nu)#	4.56 (−230.81–404.40)	−4.36 (−335.34–166.33)	0.868				
		LF (nu)#	3 **0.2**6 (17.77)	6.62 (22.17)	0.46				
		HF (nu)#	1.37 (16.44)	−2.81 (20.57)	0.327				
		Total power (nu)	44.96 (21.94)	−19.55 (15.42)	0.010*				
		LF/HF#	0.003 (1.300)	0.35 (1.85)	0.336				
Prasad et al. (29)	National Heart Institute		**Yoga group**	**Control group**	***p*** **-value**	**Secondary outcomes**	**Yoga group**	**Control group**	***p-*** **value**
		Ht(mt)	0 ± 0	0 ± 0	NA	Depression	0.35 ± 1.08	2.38 ± 1.55	<0.001
		wt(kgs)	2.60± 1.22	1.92 ± 1.11	<0.001	MACE events	non–significant		
		WC	**1.74** **±1.72**	1.04 ±1.76	0.005				
		BMI	1.04 ± 0.86	0.72 ± 0.43	0.001				
		SBP	2.78 ± 6.52	2.62 ± 5.80	0.855				
		DBP	1.60 ± 3.58	1.14 ± 3.64	0.094				
		FBS	10.17 ± 22.14	5.89 ± 11.81	0.369				
		PP	0.20 ±0.41	0.06 ± 0.18	0.002				
		HBA1c	5.54 ± 2.16	3.06 ± 2.92	<0.001				
		LDL	15.81 ± 28.50	11.73 ±19.64	0.24				
		HDL	16.78 ± 9.3	12.19 ± 10.17	0.001				
		TG	15.52 ± 10.08	9.68 ± 11.48	<0.001				
		Tch	14.44 ± 10.27	11.07 ± 9.51	0.029				
		CIMT mean	0.003 ± 0.018	0.002 ±0.008	0.764				
Hagglund et al. (30)			Yoga group	Hydro group	*p*-value		**Yoga group**	**Hydro group**	***p*** **-value**
		**Exercise capacity** (6 min walk test)	**486** **±13.3**	**488** **±11**	**0.98**	EQ−5D	0.84 ± 0.14	0.72 ±0.26	0.11
		**Muscle strength** (sit to stand test)	**25.8** **±13.2**	**21.1** **±8.2**	**0.25**	EQ VAS	77 ± 13	71 ±26	0.5
		**Blood pressure**				**KCCQ**			
		Systolic BP (mm Hg)	122 ± 17	124 ± 22	0.76	Physical Limitation	77.4 ± 19.0	73.8 ± 23.9	0.65
		Diastolic BP (mm Hg)	77 ± 11	80 ± 10	0.2				
		Pulse (beats/min)	**70** **±15**	**79** **±21**	**0.21**	**KCCQ**			
		**Clinical Variables**				Symptoms	51.4 ±20.1	56.3 ± 18.8	0.51
		Saturation %	98 ± 2	97 ± 2	0.38	Stability			
		Sensitive CRP	2.0 ±1.4	4.6 ± 5.1	0.2	Symptom	81.9 ± 21.1	84.4 ± 12.4	0.7
		NT–Pro BNP(ng/L)	1,523 ± 366	1,794 ± 1,200	0.59	Frequency			
						Symptom	79.2 ± 22.6	84.0 ± 17.2	0.51
						Burden			
						Total	80.6 ± 21.0	84.2 ± 14.0	0.51
						Symptoms			
						Self–efficacy	67.4 ± 22.3	76.0 ± 17.2	0.24
						QOL	73.1 ± 19.5	72.2 ±18.9	0.9
						Social	68.1 ± 23.4	77.6 ±21.2	0.26
						Limitation			
						Clinical	77.2± 21.8	80.8 ± 14.8	0.59
						Summary			
						Overall	73.9 ± 18.1	77.9 ±16.1	0.54
						Summary			
						PHQ−9	4.22 ±3.3	3.0 ± 1.7	0.2
						HADS–			
						(Depression)	1.9 ± 1.9	2.0 ±2.2	0.91
						HADS	2.7 ± 3.2	3.9 ±3.4	0.34
Wahlstrum et al. (31)	Hospital based(Physiotherapy clinic)	**Hemodynamic Assessment**	**Yoga group**	**Control group**	***p*** **-value**	**HR–QOL**	**Yoga group**	**Control group**	**p value**
		HR (rate/min)	61 ± 13	70 ± 19	0.024	EQ−5D VAS	80	80	0.592
		SBP(mm hg)	132 ±17	141 ±17	0.033		(50.0–100.0)	(30.0–95.0)	
		DBP(mm Hg)	77 ± 0	86 ±12	0.001	SF 36 MCS	50.6 52.70	0.016	
							(24.0–55.2)	(24.5–57.1)	
						SF 36 PCS	50.2 49.0	0.837	
							(27.6–59.1)	(29.1–61.6)	
									
Krishna et al. (32)	Medical Education &Research Institute		**Yoga group**	**Control group**	***p*** **-alue**				
		TAOS(mm)	0.99 ± 0.33	0.45 ± 0.16	HS				
		MDS(mum)	4.30 ± 1.87	9.08 ±9.08	HS				
		RER	5.00 ± 2.9	21.49 ±21.53	HS				
		hsCRP(ng/ml)	2,655.21 ± 1,286.35	8.260.47 ±2.369.90	HS				
		TNF alpha(pg/ml)	128.74 ± 43.59	185.75 ±58.17	HS				
		IL−6(pg/ml)	204.23 ±73.21	272.11 ±91.89	HS				
Raghuram et al. 2014 (33)	Hospital based	**Cardiac function**	**Yoga group**	**Control group**	***p-*** **value**		**Yoga group**	**Control group**	***p*** **-value**
		EF Total	55.91 ± 5.21	54.12 ± 6.84	0.5	PSS	15.54 ± 4.5	16.75 ± 4.30	0.12
		EF <53	53.28 ± 5.69	48.89 ± 6.76	0.02	**PANAS**			
		EF>53	58.83 ± 2.27	55.91 ± 5.21	0.38	Positive	40.54 ± 7.97	35.83 ± 8.72	0.02
		**Lipid Profile**				Negative	26.82 ± 8.08	26.3 ± 7.62	0.97
		Total cholesterol	163.04 ± 38.01	167.43 ± 38.90	0.61	**HADS**			
		Total TGLYD	142.57 ± 62.9	155.28 ± 57.98	0.03	Anxiety	5.75 ± 3.46	6.15 ± 2.98	
		Total HDL	40.23 ± 9.30	37.17 ± 9.68	0.003	Depression	4.65 ± 3.51	5.61 ± 3.30	0.07
		Total LDL	96.61 ± 29.51	98.77 ± 33.53	0.75				
		Total VLDL	28.51 ± 12.59	31.58 ± 13.22	0.03				
		BMI	23.93 ± 2.56	24.93 ± 3.46	0.001				
		Body wt(kg)	64.12 ± 7.42	67.34 ± 10.41	0.01				
		FBS Total(mg/5)	119.50 ± 45.64	124.02 ± 46.49	0.75				
Pal et al. (34)	Medical University	**Body composition**	**Yoga group**	**Control group**	***p*** **-value**				
		BMI	24.3 ± 3.5	25.1 ± 4.6	0.0,001				
		WHR	1.0 ± 0.1	1.0 ± 0.1	0.005				
		**Autonomic functions**							
		SBP	123.1 ± 9.4	129.1 ± 9.3	0.0,006				
		DBP	80.5 ± 5.1	83.8 ± 5.7	0.002				
		HR	70.5 ± 7.5	73.3 ± 8.7	0.0,006				
		Fall in SBP	10.1 ± 4.3	9.3 ± 4.2	0.38				
		Inc in DBP after	12.1 ± 4.7	10.5 ± 5.6	0.59				
		**Sustained hand grip DBP**							
		Exp Insp Ratio	1.2 ± 0.1	1.1 ± 0.2	0.44				
		30:15 beat ratio	1.1 ± 0.2	1.1 ± 0.2	0.88				
		Valsalva ratio	1.6 ± 0.9	1.5 ± 0.6	0.49				
		LF/HF ratio	1.6 ± 1.2	1.6 ± 1.2	0.27				
Pal et al. (35)	Cardiology department, Medical Institute	**Anthropometric measures**	**Yoga group**	**Control group**	**p value**				
		BMI(kg/m2)	1.45 ± 1.74	0.96 ±1.16	0.04				
		Fat %	3.09 ± 3.36	1.28 ± 2.26	0.002				
		FAT MASS	1.99 ± 2.70	2.68 ± 4.05	0.21				
		FFM	1.51 ± 6.35	0.54 ± 6.24	0.04				
		Total body water	2.47 ± 3.26	2.95 ± 4.01	0.41				
		**Blood Pressure**							
		SBP	11.02 ± 9.46	7.05 ± 6.29	0.002				
		DBP	8.85 ± 7.92	6.01 ± 4.98	0.001				
		HR	4.17 ± 10.64	2.32 ± 7.12	0.0,001				
		**Cholesterol**							
		Total cholesterol	28.29 ±30.86	5.31 ± 40.93	0.0,001				
		HDL	6.44 ± 4.92	2.0 ± 6.88	0.0,001				
		TG	38.04 ± 37.39	7.33 ± 34.82	0.0,001				
		LDL	15.10 ± 45.23	1.09 ± 34.64	0.04				
Pullen et al. (36)	Hospital based	**Cardiovascular parameters**	**Yoga group**	**Control group**	***p*** **-value**		**Yoga group**	**Control group**	***p*** **-value**
		Weight(kg)	0.63 ± 2.3	0.63 ± 3.3	0.983	MLwHFQ–T	11.56 ± 19.18	1.93 ± 16.87	0.133
		flexibility(cms)	5.0 ± 4.0	1.2 ± 4.	0.012	MLwHFQ–P	5.0 ± 8.87	0.5 ± 7.55	0.128
		GxT(s)	123 ± 108.95	116.31	0.002	MLwHFQ–E	2.35 ±7.12	0.13 ± 8.23	0.451
		Vo2 peak(ml/kg/min)	3.11 ± 3.04	3.08	0.003				
		IL−6(pg/ml)	3.57 ± 1.97	0.93	0.001				
		hs–CRP(mg/L)	0.5 ± 0.49	0.12 ± 0.17	0.001				
		EC–SOD(U/ml)	1.01 ± 73.75	18.12	0.001				
Pullen et al. (37)	Hospital based		**Yoga group**	**Control group**	***p*** **-alue**		**Yoga group**	**Control group**	***P*** **-value**
		Exercise testing	436 + 218	578 ±193	0.03	MLwHFQ–T	26.9± 16.8	41.2 ±32.1	0.643
		VO2 peak(ml/kg/min)	15.1 ± 5.21	9.7 ± 5.0	0.02	MLwHFQ–P	14 ±6.8	21 ± 113.4	0.699
		Weight(lb)	211.7 ± 56	219.5 ± 41.9	0.6	MLwHFQ–E	5 ± 4.3	12.7 ±10.8	0.774
		Flexibility(inch)	3.63 ± 3.9	3.61 ± 2.1	0.643				
		**Soluble inflammatory markers**							
		IL−6(mg/dl)	13.6± 4.5		<0.001				
		CRP(mg/dl)	1.75 ± 0.39		0.002				
		EC-SOD(U/ml)	640 ± 67		<0.001				

*LVEF; left ventricular ejection fraction; LVEDD; left ventricular end diastolic diameter; LVESD, left ventricular end diastolic diameter; HR, heart rate, DBP, diastolic blood pressure; SBP, systolic blood pressure; MET, metabolic equivalent; LVEF, left ventricular ejection fraction; TAOS, total antioxidant status; MDS, malondialdihyde, RER, redox ratio; hsCRP, high-sensitivity C-reactive protein; IL-6, interleukin-6; CDS, Cardiac Depression scale; HAM-A, Hamilton Anxiety Rating scale; DASI, Duke Anxiety Status Index; EQ-5D, EuroQoL-5D; SF-36 MCS, Short Form 36 Mental Health; SF-36 PCS, Short Form 36; VAS, visual analog scale; EQ-Euro QOL, EQ-Euro QOL 5 dimension summarized index; HADS; Hospital Anxiety and Depression Scale; KCCQ, Kansas City Cardiomyopathy Questionnaire; PHQ, Patient health questionnaire*.

## Discussion

This systematic review and meta-analysis enrolled15 RCTs examining the role of yogic interventions in managing physiological and psychosocial parameters in patients diagnosed with cardiac diseases. The heterogenous but promising results indicate significant improvements in several psychosocial outcomes including quality of life, stress and depression, and low to moderate effects on physiological parameters. The analysis reported evidence of reduction in systolic and diastolic blood pressures along with reduction in total cholesterol, low-density lipoproteins, and triglyceride levels, and increase in high-density lipoproteins levels. The results of this systematic review are in line with studies and previous reviews reporting the role of yoga in improving psychosocial and physiological outcomes in cardiac patients (5, 40). The literature reveals positive physiological effects on cardiac parameters like heart rate, lipid profile, blood pressure, respiration rate, and oxygen consumption with yogic interventions (21, 32, 41–44). Sivasankaran et al. discussed about favorable changes in endothelial-dependent vasodilatation in cardiac patients brought about by yoga and meditation (45). Yoga is known to lower heart rate and blood pressure under cardiac conditions by modulating the autonomic nervous system. Yoga and meditation have also been shown to raise melatonin, aminobutyric acid, and a variety of other neurotransmitters. Importantly, a drop in stress markers like 8-hydroxydeoxyguanosine and an increase in endorphin levels with yoga clearly imply that it can help people cope with stress (44, 45).

Many studies have evidenced the relationship between psychosocial factors and vascular functions (46, 47). Psychosocial variables like stress, depression, and anxiety are associated with cardiovascular diseases in different stages, i.e., causing arteriosclerosis and severe cardiac symptoms that may lead to development of chronic diseases (20, 48, 49). All the research studies showed that cardiac patients engage in yoga practices because of several reasons. First, the interventions are easier to learn and motivate patients to play a more active role in their own treatment. Second, after proper learning sessions, most of the exercises can be performed at home without any external assistance. Furthermore, the exercises are relatively cost-effective and involve minimal physical and emotional risks (50, 51). Despite requiring commitment and time adherence, yogic interventions are progressively gaining popularity (52).

Yoga programs have also been demonstrated to enhance physical function measures like balance, strength, and endurance, as well as symptoms in cardiac patients. Therefore, a growing body of research supports yoga's beneficial neurohumoral benefits such as lower serum cortisol, catecholamine, and aldosterone levels.

## Strengths and Limitations

The large-scale search conducted on different databases, inclusion of exclusive randomized controlled trials, and methodological quality assessment conducted the meta-analysis are the strong points of the study. Studies that had only yogic interventions were included, thus making comparison of studies feasible. Studies that included yoga as part of mind-body practices that could have brought variability between the results obtained were not included.

This meta-analysis generates evidence for the effectiveness of yoga intervention on lipid profile, blood pressure, and psychosocial outcomes in patients diagnosed with cardiac diseases. Sixteen RCTs examining the role of yogic interventions in managing physiological and psychosocial parameters in patients diagnosed with cardiac diseases were enrolled. Heterogeneity was high for most of the variables. Second, variations in types of yoga in the included articles and differences between yoga interventions, outcomes measured (physical and psychological), and variations in control groups, again possibly resulting in different effects on physiological and psychological parameters in patients with cardiac diseases, also led to heterogeneity. Therefore, high heterogeneity brought the need for more large scale high-quality RCTs to affirm the findings. Third, the included articles were limited to those published in the English language only. Some articles published in other languages might have been missed. Finally, more articles with high-level evidence such as randomized controlled trials using a consistent control group should be further conducted to assess the efficacy of yoga in patients with cardiac diseases. Therefore, in this study, publication bias cannot be ruled out because of assessment of heterogeneity and small number of studies.

## Conclusion

This systematic review and meta-analysis showed strong evidence of the effectiveness of yogic interventions in the lipid profile, blood pressure, depression, stress, anxiety, and quality of life of patients diagnosed with cardiac diseases. However, because of relatively small sample sizes of some of the trials, the results of the current review must be interpreted with caution. Randomized controlled trials with large sample sizes and rigorous study designs are required to improve our understanding of the physiological and psychosocial effects of yogic interventions on cardiac parameters. Furthermore, in the future, RCTs should address the optimal duration and frequency of yogic interventions in specific cardiac diseases so that their generalizability can be enhanced.

## Data Availability Statement

The original contributions presented in the study are included in the article/supplementary material, further inquiries can be directed to the corresponding author/s.

## Author Contributions

Conception and design of the work and drafting the work: SK, MM, PA, and RS. Substantial contributions to the acquisition of data for the work: MAlq, SP, VJ, FA, MS, and AS. Substantial contributions to the analysis of data for the work: MS, EM, MAlz, and AA. Substantial contributions to the interpretation of data for the work, final approval of the version to be published, and agreement to be accountable for all aspects of the work in ensuring that questions related to the accuracy or integrity of any part of the work are appropriately investigated and resolved: SK, MM, GS, PA, SA, HA, and SS. Revising the draft of the work critically for important intellectual content: ML, SK, AS, GS, and SA. All authors contributed to the article and approved the submitted version.

## Funding

The authors are grateful to the Deanship of Scientific Research, Majmaah University, for funding through Deanship of Scientific Research vide Project No. RGP-2019-35. The authors are also thankful to AlMareefa and Saudi Electronic University, Riyadh, Saudi Arabia, for providing support to do this research.

## Conflict of Interest

The authors declare that the research was conducted in the absence of any commercial or financial relationships that could be construed as a potential conflict of interest.

## Publisher's Note

All claims expressed in this article are solely those of the authors and do not necessarily represent those of their affiliated organizations, or those of the publisher, the editors and the reviewers. Any product that may be evaluated in this article, or claim that may be made by its manufacturer, is not guaranteed or endorsed by the publisher.
